# Medroxyprogesterone Acetate Inhibits Tumorigenesis in Mouse Models of Oviductal High-Grade Serous Carcinoma

**DOI:** 10.3390/cancers17213456

**Published:** 2025-10-28

**Authors:** Yali Zhai, Karan Bedi, Rong Wu, Ying Feng, Maranne E. Green, Celeste Leigh Pearce, Malcolm C. Pike, Eric R. Fearon, Kathleen R. Cho

**Affiliations:** 1 Department of Pathology, University of Michigan Medical School, Ann Arbor, MI 48109, USA; yaliz@umich.edu (Y.Z.); rongwu@umich.edu (R.W.); fearon@umich.edu (E.R.F.); 2 Department of Biostatistics, University of Michigan School of Public Health, Ann Arbor, MI 48109, USA; 3 Rogel Cancer Center, University of Michigan Medical School, Ann Arbor, MI 48109, USA; 4 Department of Internal Medicine, University of Michigan Medical School, Ann Arbor, MI 48109, USA; yingfeng@umich.edu (Y.F.); megreen@umich.edu (M.E.G.); 5 Department of Epidemiology, University of Michigan School of Public Health, Ann Arbor, MI 48109, USA; lpearce@umich.edu; 6 Department of Epidemiology & Biostatistics, Memorial Sloan Kettering Cancer Center, New York, NY 10065, USA; pikem@mskcc.org; 7 Department of Human Genetics, University of Michigan Medical School, Ann Arbor, MI 48109, USA

**Keywords:** high-grade serous carcinoma, medroxyprogesterone acetate, oral contraceptives, ovarian cancer prevention, genetically engineered mouse model, epithelial–mesenchymal transition

## Abstract

Background/Objectives: Tubo-ovarian high-grade serous carcinoma (HGSC) is a highly lethal malignancy, usually diagnosed at an advanced stage due to the lack of early symptoms and biomarkers. Contraceptive hormone use is associated with a reduced risk of HGSC, but the relative contributions of natural versus synthetic progestins, and their interaction with estrogens, are poorly understood. Methods: We evaluated the chemo-preventive efficacy of a synthetic progestin medroxyprogesterone acetate (MPA), progesterone (P4), and combined 17β-estradiol-progesterone (E2 + P4) in a well-characterized genetically engineered mouse model (GEMM) of oviductal HGSC based on the conditional inactivation of one or both alleles of the *Brca1*, *Trp53*, *Rb1*, and *Nf1* tumor suppressor genes (*BPRN*-het and *BPRN*-homo mice, respectively). Mice received hormones or placebo via slow-release pellets implanted subcutaneously. After induction of tumor formation, the mice were monitored for tumor development, progression, and survival. Tumor incidence was assessed histologically, and hormone effects were further explored via RNA-seq analysis of oviductal tissues. Results: MPA significantly reduced HGSC incidence and delayed tumor progression compared to the placebo, P4, and P4 + E2 in both *BPRN*-homo and *BPRN*-het mice, with up to 78% tumor-free survival in the MPA-treated *BPRN*-het cohort. P4 monotherapy did not provide significant protection vs. the placebo, but the effects of P4 could have been impacted by a failure to achieve sustained release of the hormone beyond 4–8 weeks. The E2 + P4 combination accelerated tumorigenesis and reduced survival (*p* < 0.0001 in *BPRN*-homo and *p* = 0.0004 in *BPRN*-het mice). MPA did not affect tumorigenesis in a colon cancer GEMM, or the growth of mouse HGSC-derived cells in vivo, suggesting the role of MPA in the early stages of HGSC development. Gene expression analyses showed that P4 and MPA downregulated cholesterol homeostasis, early and late estrogen response, and epithelial–mesenchymal transition pathways, though only MPA afforded tumor protection. Conclusions: These findings demonstrate that a synthetic progestin, specifically MPA, confers robust protection against HGSC development, while a combination including E2 (E2 + P4) increases risk. This work also illustrates how HGSC GEMMs can be used to compare the chemo-preventive effects of various synthetic progestins on HGSC development in order to prioritize the most effective ones for use in preventing HGSC in both general and high-risk populations.

## Simple Summary

Oral contraceptive use is known to significantly reduce the risk of developing the most common and lethal type of ovarian cancer, known as high-grade serous carcinoma (HGSC). The risk reduction is likely attributable to synthetic progestins in oral contraceptives, but it is not known which of the many types of progestins are most effective, and whether combination with estrogen mitigates their protective effects. We used genetically engineered mouse models of tubo-ovarian HGSC to show that medroxyprogesterone acetate (MPA), a common constituent of contraceptive hormones, significantly inhibits tumor development and prolongs survival. In contrast, estradiol in combination with progesterone accelerates tumorigenesis and significantly shortens survival. The findings provide strong support for MPA as a chemo-preventive agent for HGSC, confirming observations based on large-scale retrospective studies in humans. The model system offers a platform for testing other progestins and dosing strategies to identify the most effective ones in preventing HGSC in high-risk populations.

## 1. Introduction

Tubo-ovarian high-grade serous carcinoma (HGSC) is a highly aggressive and lethal type of cancer, often diagnosed at an advanced stage due to the absence of early symptoms and reliable biomarkers. Despite advancements in treatment, the prognosis for women with HGSC remains poor, with a five-year survival rate of approximately 40% [1]. Therefore, the development of effective preventive strategies is of high importance, particularly for women who are genetically predisposed to developing HGSC. Oral contraceptives are the most effective chemical drugs for preventing ovarian cancer known to date. Several epidemiological studies have reported that the use of oral contraceptives is inversely associated with ovarian cancer risk, with the protective effect increasing with duration of use [2,3,4,5]. The risk reduction does not appear to be specific to any particular formulation and is observed for all histologic types of ovarian cancer—though the risk reduction is lower for mucinous carcinoma in some studies [2,6]. A population-based study by Ness et al. found that after adjustment for current age, number of pregnancies, race, and family history of ovarian cancer, the use of low-estrogen/low-progestin oral contraceptives afforded an estimated risk reduction that was comparable to that for high-estrogen/high-progestin formulations [4]. Interestingly, a meta-analysis of 14 population-based studies found that hormone replacement therapy with unopposed estrogen increases the risk of ovarian cancer in a duration-dependent manner, while the addition of progestins at least partially blocks this effect [7]. Several questions regarding the protection afforded by oral contraceptive use remain unanswered. For example, though the protection appears to be independent of the estrogen and progestin doses, it is less clear whether progestin-only contraceptives provide a comparable level of protection to those containing both estrogen and progestin, or whether protective effects vary depending on the specific progestin used or their route of administration (oral, depot, or in intrauterine devices). Medroxyprogesterone acetate (MPA) is a synthetic progestin widely used in contraceptives and hormone replacement therapies. We have previously reported that depot-MPA use is associated with a decreased risk of ovarian cancer [8]. In contrast, natural progesterone (P4) has been proposed as a pro-tumorigenic endogenous factor in a mouse model of HGSC based on the inactivation of *Dicer1* and *Pten* [9].

In this study, we utilized well-characterized genetically engineered mouse models (GEMMs) of oviductal HGSC based on the conditional mono- or biallelic somatic inactivation of the *Brca1*, *Trp53*, *Rb1,* and *Nf1* tumor suppressor genes (TSGs) in the oviductal epithelium (*BPRN*-het and *BPRN*-homo mice, respectively) [10,11,12,13] to compare the efficacy of MPA, P4, and E2 + P4, delivered via slow-release pellets implanted subcutaneously, in preventing tumorigenesis.

## 2. Materials and Methods

### 2.1. Genetically Engineered Mice and Animal Care

The generation and characterization of *Ovgp1-iCreER^T2^* transgenic mice with various combinations of floxed TSG alleles, including *BPRN*-homo and *BPRN*-het mice, have been described previously in detail [10,11]. *CDX2P-CreER^T2^*; *Apc^fl/+^*; *Kras^LSL-G12D/+^*; *Trp53^fl-R270H/fl^* mice (referred to hereafter as *AKP* mice) have also been previously reported [14]. Genotypes of the individual mice included in this study were confirmed by polymerase chain reaction (PCR) analysis of tail-derived DNA. The mice were housed in standard housing and fed the same chow diet. All procedures involving mice for the research described herein have been approved by the University of Michigan’s Institutional Animal Care and Use Committee (PRO00008343, PRO00010212, and PRO00012388).

### 2.2. In Vivo Induction of Oviductal Tumors and Implantation of Hormone Pellets

Female 6- to 8-week-old *Ovgp1-iCreER^T2^*; *BPRN*-homo, and *BPRN*-het mice were implanted with slow-release (90 day) hormone pellets subcutaneously under the dorsal skin. The placebo (25 mg), MPA (25 mg), and E2 + P4 (0.05 mg 17β-estradiol + 25 mg P4) pellets were obtained from Innovative Research of America (Sarasota, FL, USA). Slow-release (90 day) P4 (25 mg) pellets were obtained from Belma Technologies (Liege, Belgium). Groups of *BPRN*-homo mice were implanted with placebo (n = 22), P4 (n = 21), MPA (n = 24), or E2 + P4 (n = 27) pellets and 72 h later were treated transiently with Tamoxifen (TAM, 0.2 g/kg body weight, injected intraperitoneally (i.p.) on day 1 and day 3) as described previously to induce Cre-mediated recombination of the floxed TSG alleles in the oviductal epithelium. Groups of *BPRN*-het mice were similarly implanted with placebo (n = 28), P4 (n = 24), MPA (n = 25), or E2 + P4 (n = 27) pellets, and tumors were then induced with TAM as described above. After 90 days, fresh pellets were implanted for a total of 180 days of hormone treatment. Similarly, 2- to –3-month-old *AKP* mice (5 males and 5 females per group) were implanted with either placebo or MPA pellets as described above. Subsequently, the *AKP* mice received tamoxifen (TAM) at 70 mg/kg daily for two consecutive days via i.p. injection. All mice were euthanized upon reaching predefined humane endpoints or at the end of the specified monitoring period.

### 2.3. Measurement of Hormone Levels

To determine whether the pellets released the hormones as expected, serum samples were collected from 3 mice in each group (placebo, P4, and E2 + P4) via tail vein at 1 week, 4 weeks, 8 weeks, and 12 weeks after pellet insertion. P4 and E2 levels were tested by the University of Virginia Center for Research in Reproduction (Charlottesville, VA, USA). To meet the larger sample size requirement for MPA assays, we collected plasma samples from 3 to 4 mice euthanized at weeks 2 and 8 in the MPA and placebo groups. At the end of the surveillance period, we also collected plasma samples from 3 euthanized mice. MPA levels were analyzed by the Pharmacokinetics Core in the College of Pharmacy at the University of Michigan.

### 2.4. Tissue Sampling, Histopathological and Immunohistochemical Analysis

Mice in the *BPRN*-homo and *BPRN*-het cohorts were euthanized at 52 weeks and 60 weeks post-TAM, respectively, or were sacrificed earlier if they reached humane endpoints. Necropsies were performed to determine the presence and extent of disease in hormone- vs. placebo-treated mice as previously described [11,13]. Briefly, mice were grossly inspected for oviductal tumors, metastatic disease, and ascites. Bilateral ovaries and oviducts, uterus, mesentery, omentum, pancreas, kidneys, liver, and lungs were harvested from each case. Representative formalin-fixed paraffin-embedded (FFPE) sections of each tissue were stained with hematoxylin and eosin (H&E) and examined by light microscopy. Oviducts without gross lesions were examined in their entirety by exhaustive sectioning and microscopic evaluation of alternating sections. The histology of each lesion was reviewed by two participants with expertise in gynecologic pathology. Lesions were classified as serous tubal intraepithelial carcinoma (STIC), early (invasive but confined to the oviduct) HGSC or carcinosarcoma (eHGSC or eCaSa), or as invasive HGSC or carcinosarcoma extending beyond the oviduct (HGSC or CaSa), including metastasis.

### 2.5. Cell Line Establishment and Cell Culture

2D tumor-derived cell cultures were generated following the protocols described by Shepherd and colleagues [15]. Briefly, the cell line *BPRN*-3867 was derived from an oviductal tumor arising in a *BPRN*-homo mouse and then transferred to monolayer cell culture; cells were maintained in DMEM/F12 (Gibco, Fisher Scientific, Waltham, MA, USA) supplemented with 10% heat-inactivated fetal bovine serum (FBS, HyClone, Logan, UT, USA) and penicillin–streptomycin (Gibco, Fisher Scientific).

### 2.6. Allografts and MPA Treatment

*BPRN*-3867 cells were used to generate tumor allografts to test the effects of MPA on established tumors. A total of six immunocompromised, 6-week-old Nod-SCID-IL2Rgamma (NSG) mice were divided into three groups. On day 0, two mice were implanted with placebo pellets (group 1) and two mice were implanted with MPA pellets (group 2). Two days later, all six mice were subcutaneously injected in the bilateral flanks with 2 × 10^6^ cultured cells resuspended in 100 μL cold PBS and 100 μL Matrigel (Corning, New York, NY, USA). On day 9, the two mice not implanted earlier were implanted with MPA pellets (group 3). All mice were sacrificed and necropsied 60 days after cell injection. Tumor weights and volumes were obtained, with tumor volume estimated by the formula L × W × H/2 (cm^3^). The excised tumors were then fixed in 10% buffered formalin, paraffin-embedded, and sectioned for histopathological examination.

### 2.7. Gene Expression

RNA sequencing (RNA-seq) was used to profile gene expression in the following mouse oviductal epithelial tissues: (1) no treatment (no pellets, no TAM, n = 4), (2) TAM only (no pellets, n = 4), (3) placebo pellets + TAM (n = 3), (4) P4 pellets + TAM (n = 3), (5) MPA pellets + TAM (n = 3), and (6) E2 + P4 pellets + TAM (n = 3). Slow-release pellets containing steroid hormones (MPA, P4, or E2 + P4) or placebo were implanted subcutaneously into 6- to 8-week-old female *BPRN*-het mice. Three days later, the mice received intraperitoneal (i.p) TAM injections to induce Cre expression. Mice were euthanized 14 days post-TAM, and the distal portion of the oviducts were collected for RNA isolation. RNA was extracted using the miRNeasy Mini Kit (Qiagen, Germantown, MD, USA). Library prep and RNA sequencing was carried out in the Advanced Genomics Core at the University of Michigan. Subsequently, 151 bp paired-end sequencing was performed according to the manufacturer’s protocol (Illumina NovaSeq San Diego, CA, USA). The BCL Convert Conversion Software v4.0 (Illumina) was used to generate de-multiplexed Fastq files. Initial data analysis was performed by the University of Michigan Bioinformatics Core. Raw reads were trimmed using Cutadapt (v2.3) [16]. FastQC (v0.11.8) was used to ensure that only high-quality data were used for expression quantity and differential expression [17]. Fastq Screen v was used to screen for various types of contamination [18]. Reads were mapped to the reference genome GRCm38 (ENSEMBL), using STAR v2.7.8a [19] and assigned count estimates to genes with RSEM v1.3.3 [20]. Alignment options followed ENCODE standards for RNA-seq (https://github.com/alexdobin/STAR/blob/master/doc/STARmanual.pdf accessed on 23 September 2023). Multiqc v1.7 compiled the results from several of these tools and provided a detailed and comprehensive quality control report [21].

Differential gene expressions across groups were analyzed with DESeq2 in R. For enrichment tests, we mapped mouse genes to human homologs using biomaRt ensembl version 100. The obtained differentially expressed genes (DEGs, defined as fold change (FC) ≥1.5 and FDR-adjusted *p* value ≤ 0.05) were used for gene set enrichment analysis with a web-based functional enrichment analysis tool called WebGestalt (WEB-based GEne SeT AnaLysis Toolkit) available at http://www.webgestalt.org/. Gene counts produced by RSEM and the raw sequencing data are available in the NCBI’s Gene Expression Omnibus (GEO) database under the accession number GSE309487.

### 2.8. Statistical Analysis

The survival rates of tumor-bearing placebo- and hormone-treated mice were estimated by Kaplan–Meier analysis using the GraphPad Prism software version 10. The Cochran–Mantel–Haenszel Chi-square test of association was performed using SPSS 9.0 to evaluate the effects of each type of hormone on tumor incidence and progression compared to the placebo. Differences at *p* < 0.05 were considered statistically significant.

## 3. Results

### 3.1. MPA Inhibits HGSC Tumorigenesis in BPRN-homo and BPRN-het Mice

**Figure 1:**
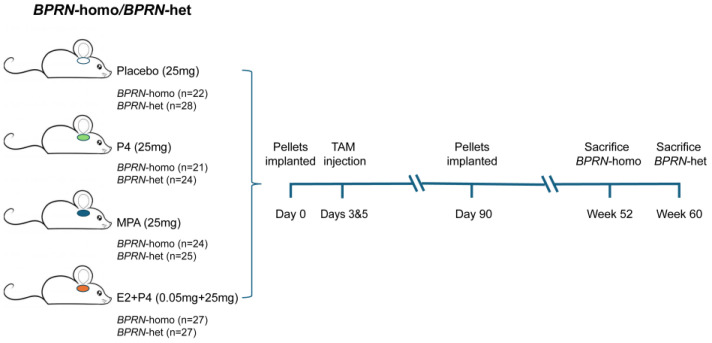
Schematic diagram of experimental design. Groups of *BPRN*-homo and *BPRN*-het mice (21–28 mice per group) were implanted with hormone or placebo pellets and monitored for at least one year after the induction of tumor formation with TAM injection.

A schematic diagram of the overall experimental design is provided in Figure 1 and representative photomicrographs of oviductal tumors that arose in our GEMMs are shown in Supplemental Figure S1.

**Figure 2:**
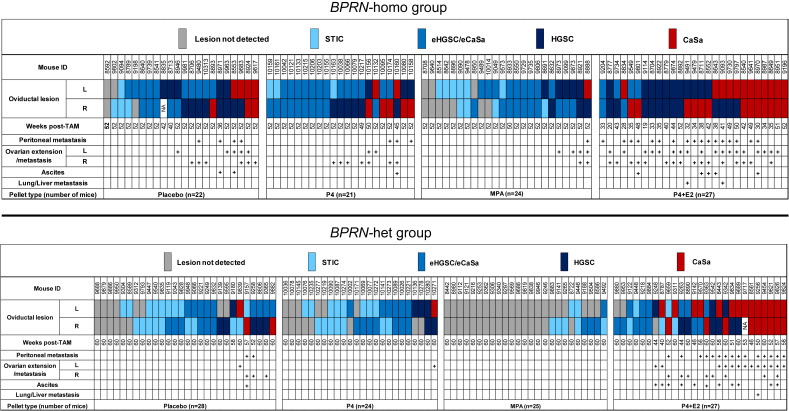
Summary of tumor development in placebo-, P4-, MPA-, and E2 + P4-treated *Ovgp1-iCreERT2*; *BPRN*-homo (**top**) and *Ovgp1-iCreERT2*; *BPRN*-het (**bottom**) mice. Weeks post-TAM indicates the time point at which the mice were euthanized. Oviductal lesions are indicated by colored boxes as indicated: serous tubal intraepithelial carcinoma (STIC); early invasive HGSC or carcinosarcoma (CaSa) confined to the oviduct (eHGSC/eCaSa); and invasive HGSC or CaSa extending beyond the oviduct.

Tumor development in cohorts of TAM-treated *BPRN* mice after the implantation of placebo, P4, MPA, and E2 + P4 pellets in *BPRN*-homo and *BPRN*-het mice are summarized in the upper and lower panels of Figure 2, respectively.

Compared with mice treated with placebo, P4 alone, or E2 + P4, we found that both *BPRN*-homo and *BPRN*-het mice treated with MPA showed significantly fewer and less advanced oviductal tumors. Specifically, in *BPRN*-homo mice 52 weeks after TAM injection, 10 of 48 (21%) oviducts in MPA-treated vs. 4 of 43 (9%) oviducts of placebo-treated mice were free of detectable tumors (Table 1). The preventive effect of MPA was even more pronounced in the *BPRN*-het mice 60 weeks after TAM injection. In these mice, 39 of 50 (78%) MPA-treated vs. 14 of 56 (25%) placebo-treated oviducts were tumor-free (Table 2). In addition, tumor progression was significantly slowed in the MPA-treated groups, with no advanced HGSCs or carcinosarcomas (CaSas) observed in *BPRN*-het mice (0% MPA vs. 16% placebo), and fewer HGSCs and CaSas observed in the *BPRN*-homo mice (19% MPA vs. 47% placebo). Statistical analysis by the Cochran–Mantel–Haenszel Chi-square association test showed significant differences in tumor incidence and progression between the MPA and placebo-treated groups in both *BPRN*-het and *BPRN*-homo mice, with significantly fewer and less advanced tumors in both MPA-treated groups compared to the placebo-treated groups (*p* values of <0.001 and *p* = 0.004, respectively). Our data shows that the synthetic progestin MPA is highly effective at inhibiting or delaying oviductal HGSC development in our GEMMs.

### 3.2. Tumor Development in BPRN-homo and BPRN-het Mice Treated with P4 Alone Is Comparable to Placebo

While MPA markedly inhibited the development and/or progression of HGSC in our GEMMs, P4 did not show a similar protective effect. As shown in Figure 2 and Table 1 and Table 2, the incidence of oviductal lesions in P4-treated *BPRN*-homo and *BPRN*-het mice was similar to that in the placebo-treated control groups (*p* = 0.48 and *p* = 0.56, respectively).

### 3.3. Progesterone (P4) in Combination with Estradiol (E2) Accelerates BPRN Tumor Development

**Figure 3:**
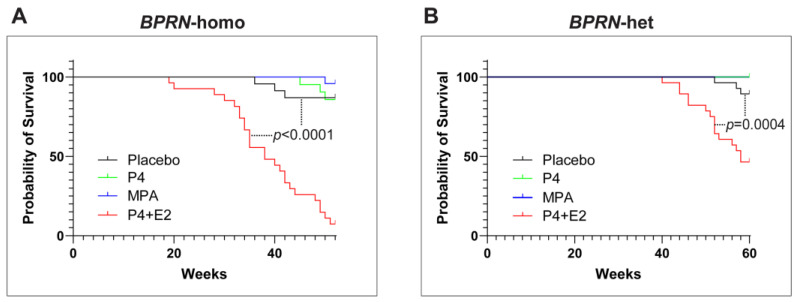
Kaplan–Meier survival curves of placebo- (black), P4- (green), MPA- (blue), and E2 + P4- (red) treated Ovgp1-iCreERT2; BPRN-homo (**A**) and Ovgp1-iCreERT2; BPRN-het (**B**) mice after TAM injection. *p* < 0.0001 when comparing the BPRN-homo E2 + P4 mice with the placebo mice (median survival, 38.8 weeks vs. 50.4 weeks) and *p* = 0.0004 when comparing the BPRN-het E2 + P4 mice with the placebo mice (median survival, 55 weeks vs. 59.5 weeks) using the log-rank (Mantel–Cox) test.

Interestingly, the combination of P4 with E2 appeared to accelerate the initiation and progression of HGSC in both *BPRN*-homo and *BPRN*-het mice (Figure 2). Among *BPRN*-homo mice receiving the E2 + P4 combination, 87% developed advanced HGSC or CaSa, compared to only 47% of the placebo-treated mice (Table 1). Similarly, 60% of E2 + P4-treated *BPRN*-het mice developed advanced HGSCs or CaSas, compared to only 16% of the placebo-treated mice. Differences in the presence and extent of tumors between the E2 + P4- and placebo-treated mice in both the *BPRN*-homo and *BPRN*-het groups were highly significant (*p* < 0.001 and *p* < 0.001, respectively). In addition, the Kaplan–Meier survival analysis showed that after E2 + P4 treatment, the survival of mice in the *BPRN*-homo and *BPRN*-het groups was significantly shorter than that of mice in the placebo control groups (median survival, *BPRN*-homo: E2 + P4 38.8 weeks vs. placebo 50.4 weeks, *p <* 0.0001, log-rank Mantel–Cox test (Figure 3A); and *BPRN*-het: E2 + P4 55 weeks vs. placebo 59.5 weeks, *p =* 0.0004 (Figure 3B)). These findings suggest that E2 has tumor-promoting effects in these GEMMs that are not ameliorated by the addition of P4.

### 3.4. Some Commercially Available Slow-Release Pellets Have Suboptimal Performance

To monitor the effectiveness of the slow-release pellets used in this study, hormone levels were measured at a series of time points after the implantation of pellets intended to consistently release P4, MPA, or E2 + P4 hormones over a period of approximately 90 days after subcutaneous implantation in the mice. As shown in Supplemental Figure S2 (left panel), the estradiol (E2) levels in mice implanted with E2 + P4 pellets peaked at week 1, had declined by week 4, and were comparable to baseline levels at week 8. Serum progesterone levels in the P4 and E2 + P4 treatment groups also peaked at week 1, then decreased significantly by week 4, and further decreased to the same level as in the placebo group by week 8 (Supplemental Figure S2, center panel). Based on these findings, it is possible that more sustained delivery of P4 could have resulted in either tumor-promoting or tumor-inhibiting effects, and our failure to observe an impact of P4 alone on tumorigenesis should be interpreted with caution. Interestingly, even short (4–8 week) exposure to E2 in combination with P4 had striking tumor-promoting effects. Plasma levels of MPA were quite consistent between mice, persisted longer, and remained elevated, even at the study endpoint (52–60 weeks, Supplemental Figure S2, right panel).

### 3.5. MPA Has No Effect on Tumorigenesis in a Colon Cancer GEMM

To evaluate the specificity of the effects of MPA on oviductal tumorigenesis, we tested the effects of MPA in a GEMM of colon cancer based on the conditional somatic inactivation of the *Apc* and *Trp53* tumor suppressor genes and activation of *Kras* (*AKP* mice) in the colonic epithelium [14]. Slow-release (90 day) MPA or placebo pellets were subcutaneously implanted into cohorts of male and female *AKP* mice and three days after pellet implantation, the mice were injected with TAM on two consecutive days to induce colonic tumor formation. In this mouse colon carcinoma model, we did not observe any difference in survival between the MPA- and placebo-treated groups of male or female mice (Supplemental Figure S3). This finding supports the conclusion that the effects of MPA are specific to certain tumor types rather than influencing tumorigenesis more broadly.

### 3.6. MPA Has No Effect on the Growth of Established HGSCs

**Figure 4:**
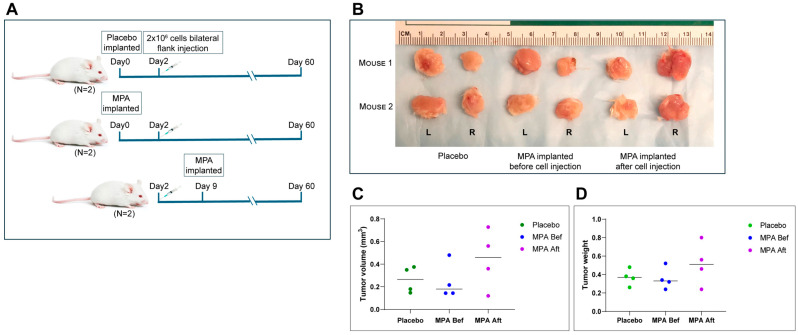
In vivo treatment of allografted tumors using MPA pellets. (**A**) Schematic diagram of experimental design. Three groups of mice (2 mice per group) were injected with *BPRN*-3867 tumor-derived cells before (Bef)/after (Aft) MPA or placebo pellet implantation and analyzed. (**B**) Images of tumors resected from each group at the study endpoint. L: left flank tumor and R: right flank tumor. (**C**) Tumor volumes in each group, measured using a vernier caliper. (**D**) Tumor weights in each group.

Based on the data shown above, *BPRN*-homo and *BPRN*-het mice treated with MPA during the period when oviductal tumors are developing show marked reduction in their numbers and extents, but it is unclear if MPA inhibits tumor initiation, progression, or both. To test MPA’s effect on established HGSCs, MPA or placebo pellets were subcutaneously implanted into NSG mice before or after injection of a tumorigenic cell line (*BPRN*-3867) derived from an oviductal tumor that arose in a TAM-treated *BPRN* mouse. MPA failed to exhibit any in vivo anti-tumor activity, regardless of whether MPA treatment was started before or after tumor cell injection (Figure 4A–D). These results indicate that MPA does not affect the growth of fully transformed cells or inhibit their ability to establish tumor allografts. Furthermore, the findings suggest that MPA is more likely to exert its effects in the early stages of tumorigenesis, either on the oviductal epithelium itself, through effects on non-neoplastic cells in the oviductal microenvironment, or both.

### 3.7. Identification of Differentially Expressed Genes

**Figure 5:**
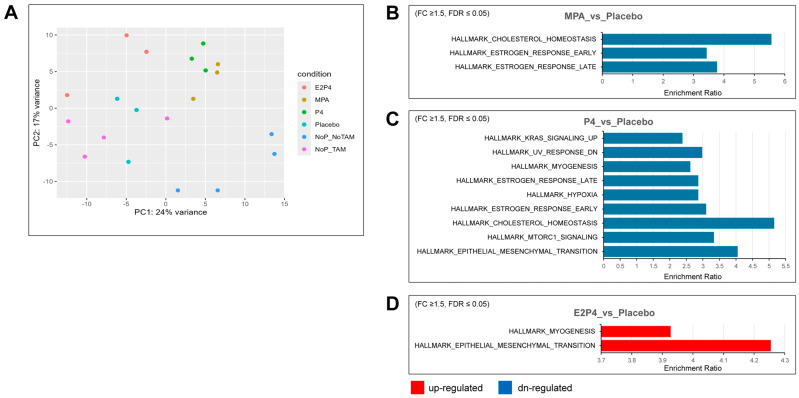
Hallmark gene sets enriched for differentially expressed genes in the MPA, P4, and E2 + P4 groups compared with the placebo group. (**A**) Global gene expression analyses were performed on oviductal tissue RNA from E2P4, MPA, P4, placebo (n = 3 for each group), and no pellets (NoP, n = 4) mice treated with TAM for 14 days and on oviductal tissue RNA from no TAM/no pellet mice (n = 4). Principal components analysis showed that the MPA group and P4 group had close global gene expression pattens, whereas the no pellet/no TAM group had a global gene expression pattern different from that of the other groups. (**B**,**C**) Downregulated (blue) Hallmark gene sets overrepresented in MPA and P4 compared with the placebo group (fold change ≥ 1.5, FDR ≤ 0.05). (**D**) Upregulated (red) Hallmark gene sets overrepresented in E2 + P4 compared with the placebo group (fold change ≥ 1.5, FDR ≤ 0.05).

To further examine the effects of each type of hormone on mouse oviductal tissue in the earliest phases of tumor development, RNA-Seq analysis was used to compare gene expression in the oviducts of *BPRN*-homo mice treated with placebo, MPA, P4, or E2 + P4 (n = 3 for each group) or no pellets (n = 4) 14 days after the induction of tumor formation via TAM injection. A group of mice (n = 4) with no TAM injection and no pellet implantation was also included. Principal components analysis showed that the MPA and P4 groups had similar global gene expression, which was distinct from the E2 + P4 and placebo groups (Figure 5A). Based on differential gene expression analysis (FDR-adjusted *p* ≤ 0.05, fold change ≥1.5 in either direction), gene set enrichment testing was performed using differentially expressed genes (DEGs) against curated Hallmark gene sets from MSigDB. Compared with the placebo group, there were three gene sets in the gene list of downregulated genes in the MPA group (Figure 5B), nine gene sets in the gene list of downregulated genes in the P4 group (Figure 5C), and two gene sets in the gene list of upregulated genes in the E2 + P4 group (Figure 5D).

**Figure 6:**
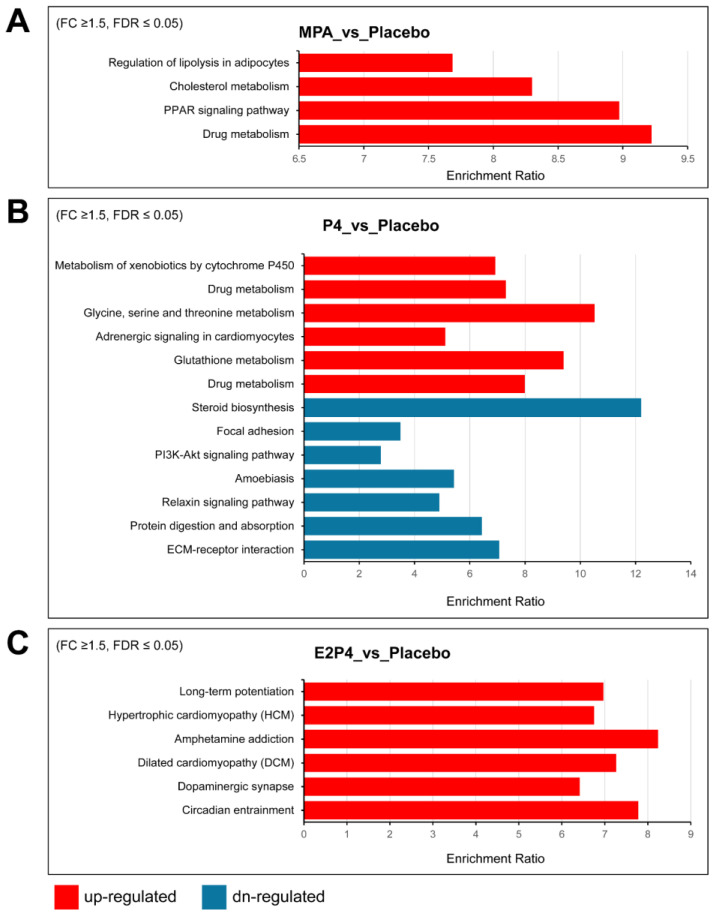
KEGG pathway analysis of differentially expressed upregulated and downregulated genes in oviductal tissues of BPRN-conditional knockout mice. (**A**) KEGG pathways overrepresented in the lists of genes upregulated (red) in mouse oviductal tissues of MPA-treated mice compared with placebo mice after 14 days of TAM treatment. (**B**) KEGG pathways overrepresented in the lists of genes up- (red) and downregulated (blue) in mouse oviductal tissues of P4-treated mice compared with placebo mice after 14 days of TAM treatment. (**C**) KEGG pathways overrepresented in the lists of genes upregulated (red) in mouse oviductal tissues of E2P4-treated mice compared with placebo mice after 14 days of TAM treatment. The enriched pathways with FDR ≤ 0.05 are shown.

Supplemental Table S1 lists additional upregulated and downregulated Hallmark gene sets enriched for differentially expressed genes, providing more gene sets with trend changes. Notably, consistent with the aggressiveness of the E2 + P4 group, the most enriched upregulated gene set was the epithelial–mesenchymal transition (EMT) set, with 13 of 200 EMT genes, including *COL11A1*, *ECM1*, *TGFBI*, *BGN*, *TNFRSF11B*, *TNFRSF12A*, and *TIMP1*. Furthermore, several matrix metalloproteinases were also upregulated in the E2 + P4 group, including *MMP7*, *MMP13*, and *MMP19*. In contrast, we observed downregulation of the EMT gene set in the P4 group (17 of 200 EMT genes) including *CCN1*, *CDH11*, *SERPINE1,* and some types of collagens (*COL1A1*, *COL1A2*, *COL3A1*, *COL4A2,* and *COL6A2*). A trend for downregulation of the EMT gene set was also noted in the MPA group (*p* = 0.0079925, FDR = 0.067), including several downregulated EMT genes (*CCN1*, *COL1A1*, *COL1A2,* and *COL3A1*). In addition, both the P4 and MPA groups had downregulation of Hallmark gene sets for estrogen response and cholesterol homeostasis, indicating that MPA and P4 share some similarities in regulating gene expression. KEGG pathway analysis of differentially upregulated and downregulated genes identified some differences between the MPA and P4 groups vs. the placebo group that may be related to the tumor-inhibiting effects of MPA but not P4 (Figure 6). Specifically, regulation of lipolysis in adipocytes, cholesterol metabolism, and PPAR signaling were upregulated by MPA treatment, but not by P4.

Lists of the 50 most upregulated and downregulated genes in the placebo-treated mice vs. mice treated with P4, MPA, or E2 + P4 are provided in Supplemental Tables S2, S3, and S4, respectively.

## 4. Discussion

In this work, we showed that medroxyprogesterone acetate significantly reduced tumor incidence and delayed tumor progression in mouse models of oviductal high-grade serous carcinoma. The preventive effect of MPA was more pronounced in *BPRN*-het than in *BPRN*-homo mice. This latter finding is consistent with prior work using these model systems, which suggested the impact of interventions during the long (several month) latency period between tumor induction and emergence of early oviductal lesions is more apparent in mice with fewer initially targeted gene mutations. For example, we found that a preventive effect of multiparity was seen in *BPRN^fl/+^* mice (carrying two floxed alleles of *Brca1*, *Trp53,* and *Rb1*, but only one floxed *Nf1* allele), but not in *BPRN*-homo mice [12]. In both *BPRN*-het and *BPRN*-homo mice, an anti-tumor effect was not observed with P4 alone, and addition of 17β-estradiol to P4 significantly enhanced tumorigenesis and reduced survival in both model systems. While the protective versus deleterious impact of MPA and E2, respectively, in our GEMMs are concordant with large-scale epidemiological studies in humans [7,8,22], some limitations of our study are acknowledged. First, our GEMMs are based on simultaneous mono- or biallelic inactivation of four TSGs that are not broadly representative of human HGSCs, including those that are HRD-proficient. While all four of these TSGs are on the list of genes with statistically recurrent somatic alterations in the HGSC Cancer Genome Atlas [23], human tumors do not arise via simultaneous inactivation of all four TSGs. Second, given the length of time and expense associated with testing the effects of hormones as chemo-preventive agents in our GEMMs, only a limited number of chemo-preventive regimens could be tested in this initial study. Third, mice are not humans and may not have the same pharmacodynamics with respect to hormone dosing and tissue response. Finally, our selection of hormones and hormone combinations was impacted by what is commercially available (alone and in combination) for use in mice and, as noted above, some of the pellets did not consistently release hormones over the designated 90-day period. As a consequence, the effects of P4 and E2 + P4 may have been significantly influenced by shorter and lower dosing than we anticipated. Nonetheless, we found that MPA alone had a robust preventive effect on oviductal tumorigenesis and even intermittent exposure to E2 + P4 accelerated tumorigenesis in our model systems. Notably, a large-scale clinical trial assessing the long-term influence of estrogen alone or in combination with MPA for menopausal hormone therapy found that estrogen alone increased ovarian cancer incidence and mortality, while estrogen plus MPA did not [24]. Similarly, in pooled data from case–control studies, Lee and colleagues found that estrogen in combination with a progestin for menopausal therapy was not associated with an increased risk of ovarian cancer, but information on the progestin dose and type was not available for analysis [25]. Unfortunately, we were not able to test the effects of E2 + MPA in our model system because pellets with that hormone combination are not readily available.

The mechanisms by which MPA confers protection from oviductal HGSC development remain unclear. MPA blocks follicular maturation and ovulation, thickens the cervical mucus to impede sperm motility, and renders the endometrium less favorable for implantation [26]. MPA may also have direct effects on the oviductal/fallopian tube epithelium itself, but the protective effect of MPA does not appear to be due to nonspecific repression of cell proliferation, as MPA did not suppress the growth of established *BPRN* HGSC allografts or alter the proliferative index of oviductal epithelium based on Ki-67 expression (Cho lab, unpublished data), nor did it inhibit tumor development or progression in a mouse colon cancer GEMM. These findings, along with the absence of any detectable lesions in the majority of the MPA-treated *BPRN*-het mice, suggest that MPA acts on specific hormone-responsive cell types primarily at the initiation or pre-malignant transformation stages of tumorigenesis, perhaps by altering the fate of at-risk oviductal epithelial cells or by modifying the tissue microenvironment in a manner that is unfavorable for malignant transformation. Interestingly, Nelson and colleagues investigated the effects of depo-medroxyprogesterone acetate (DMPA) on the development of fallopian tube/oviductal carcinomas in a different transgenic mouse model of oviductal HGSC (Mogp-Tag mice) [27]. In this model, mice treated with DMPA accumulated fewer p53 positive cells, p53 signature lesions, serous tubal intraepithelial carcinomas (STICs), and adenocarcinomas in their oviducts. DMPA was found to induce cleaved caspase-3 in the fallopian tube/oviductal epithelium, suggesting the involvement of apoptosis in DMPA-related clearance of genetically damaged cells in the oviduct. Like us, these investigators suggest that progestins target early rather than late events in the development of HGSC. While our gene expression analysis of hormone-exposed oviductal epithelium from *BPRN* mice did not identify apoptosis pathway genes differentially expressed in the MPA- vs. placebo-treated oviductal epithelium, our data suggest possible roles for MPA in cholesterol metabolism and the downregulation of EMT genes, either in the oviductal epithelium, other cells in the oviductal microenvironment during tumor development, or both. While MPA and P4 have similar effects on EMT signaling, cholesterol homeostasis, and estrogen-responsive gene sets, their different effects on tumor development and progression suggest that there are important distinctions between synthetic and natural progestins in terms of receptor activity or downstream signaling, which need further investigation. Interestingly, simvastatin, a drug that lowers cholesterol levels by inhibiting a key enzyme in cholesterol synthesis (HMG-CoA reductase), has been shown to interfere with cancer stem cell plasticity and reduce metastasis in ovarian cancer [28], and statin use has been associated with a lower risk of both serous and non-serous types of ovarian carcinoma [29].

Our findings regarding the E2 + P4 combination are quite striking, as the combination exhibited a strong effect in accelerating HGSC development in *BPRN* mice, which is consistent with epidemiological data showing that estrogen therapy alone or in combination is a risk factor for ovarian cancer [7,24,25]. Unfortunately, we were unable to include an E2-only cohort of mice in this study due to the failure of the E2 pellets we tested to release the hormone in a suitable fashion. Notably, our gene expression analysis revealed that EMT was the most highly enriched Hallmark gene set upregulated by E2 + P4 compared to placebo, while EMT pathway genes were downregulated by P4 and MPA. Previous studies have shown that estrogen can promote EMT through the estrogen receptor, downregulating E-cadherin and upregulating Snail and vimentin in breast and prostate cancer cells [30,31,32]. Wang and colleagues also demonstrated that estrogen can promote EMT in a subset of *Brca1*-deficient breast cancer cells through an ER-independent signaling pathway [33]. Estrogen’s interactions with its receptor are also known to regulate the expression and activity of various MMPs, some of which are known to be associated with EMT and promote tumor growth and metastasis [34,35,36,37]. Our results are concordant with those of Laviolette et al., who employed yet another transgenic model of ovarian cancer (tgCAG-LS-Tag mice) to show that estrogen promoted tumor development and progression while P4 had no impact [38]. In contrast, another study reported that in the *Dicer1 ^flox/flox^ Pten ^flox/flox^ Amhr2* ^cre/+^ ovarian cancer GEMM, treatment with progesterone (P4) alone significantly accelerated tumor development and progression in all ovariectomized *Dicer1-Pten* double-knockout (DKO) mice, whereas the addition of combined estrogen and progesterone treatment inhibited this rapid progression [9]. These observations highlight the importance of hormone balance, timing, and receptor context in mediating cancer risks. The selected engineered alleles, specificity of the induced genetic alterations to the oviductal epithelium, and genetic background in different mouse models are undoubtedly influencing factors.

The relationship between hormones and ovarian cancer is complex and depends on many factors, including the type and duration of hormone exposure, individual genetics and medical history, and the specific subtype of ovarian cancer. Our ability to test and compare the chemo-preventive effects of specific natural and synthetic hormones and hormone combinations in a controlled genetic context and well-characterized model system represents a significant advance. Our work supports the use of synthetic progestins in chemo-preventive regimens aimed at reducing the risk of tubo-ovarian HGSC and perhaps other types of ovarian carcinomas. What is less clear is whether some progestins are more effective than others at reducing risk and whether the magnitude of risk reduction may be related to the type of progestin, dose, and/or dosing method. Importantly, as noted by Stanczyk over two decades ago, all progestins are not created equal [39]. Some are structurally related to progesterone (including MPA), while others are structurally related to testosterone (such as norethindrone and levonorgestrel). Moreover, the various progestins used in oral contraceptives and hormone replacement therapies have differences in metabolism, pharmacokinetics, receptor interactions, intracellular actions, and biological effects that may impact their efficacy as chemo-preventive agents for HGSC [40]. Given the popularity of hormone-releasing intrauterine devices for contraception, our model system could be used to test whether the progestins in these devices (e.g., levonorgestrel) confers comparable anti-tumor protection to MPA. Similar studies could be performed with norethindrone and other common constituents of contraceptive medications that may be equally efficacious for contraception but have different impacts on reducing the risk of developing one of the most lethal cancers affecting women.

## 5. Conclusions

The current study illustrates the utility of high-fidelity mouse models of HGSC in testing various chemoprevention strategies that could reduce the risk of HGSC in both the general population and genetically predisposed women. Our findings provide experimental evidence confirming a robust protective effect of medroxyprogesterone acetate and supporting a pro-tumorigenic effect of estradiol. The GEMMs offer a suitable platform for testing other progestins and dosing strategies to identify the most effective ones in preventing HGSC.

## Figures and Tables

**Table 1:** Comparison of oviductal HGSC development in hormone-treated *BPRN*-homo mice.

Case/Total (%)					
Cohorts	No Lesion	STIC	eHGSC/eCaSa	HGSC/CaSa	*p* Value
Placebo	4/43 (9.3)	4/43 (9.3)	15/43 (34.88)	20/43 (46.51)	
P4	0/42 (0.0)	3/42 (7.14)	23/42 (54.8)	16/42 (38.1)	=0.48
MPA	10/48 (20.8)	10/48 (20.8)	19/48 (39.58)	9/48 (18.75)	=0.004 *
E2 + P4	0/54 (0.0)	0/54 (0.0)	7/54 (12.96	47/54 (87.04)	<0.001 ***

*, *p* < 0.05 and ***, *p* < 0.001 versus control; Cochrane–Mantel–Haenszel Chi-square test of association. Numbers shown reflect two oviducts (right and left) for each mouse included in the analysis.

**Table 2:** Comparison of oviductal HGSC development in the hormone-treated *BPRN*-het mice.

Case/Total (%)					
Cohorts	No Lesion	STIC	eHGSC/eCaSa	HGSC/CaSa	*p* Value
Placebo	14/56 (25.0)	18/56 (32.14)	15/56 (26.79)	9/56 (16.07)	
P4	14/48 (29.17)	15/48 (31.30)	13/48 (27.1)	6/48 (12.5)	=0.58
MPA	39/50 (78.0)	7/50 (14.0)	4/50 (8.00)	0/50 (0.00)	<0.001 ***
E2 + P4	3/53 (5.7)	5/53 (9.43)	13/53 (24.52)	32/53 (60.38)	<0.001 ***

***, *p* < 0.001 versus control; Cochrane–Mantel–Haenszel Chi-square test of association. Numbers shown reflect two oviducts (right and left) for each mouse included in the analysis.

## Data Availability

The original RNA-seq data presented in the study are openly available in the NCBI’s Gene Expression Omnibus (GEO) database under the accession number GSE309487. The remaining original contributions presented in this study are included in the article/Supplementary Material. Further inquiries can be directed to the corresponding author.

## Supplementary Materials

The following supporting information can be downloaded at https://www.mdpi.com/article/10.3390/cancers17213456/s1. Figure S1: Representative photomicrographs of H&E-stained sections showing progression of oviductal lesions in *BPRN*-homo mice. Figure S2: Serum/plasma hormone levels (E2, P4, and MPA) in *BPRN*-homo mice at different time points with P4, MPA, and E2 + P4 treatment. Figure S3: Kaplan–Meier survival curves of TAM-treated *AKP* mice implanted with pellets containing MPA vs. placebo. Table S1: Hallmark gene sets enriched with differentially expressed genes in the E2 + P4 and MPA groups compared with the placebo group. Table S2: Top 50 upregulated and 50 downregulated genes in the oviducts of P4- vs. placebo-treated mice. Table S3: Top 50 upregulated and 50 downregulated genes in the oviducts of MPA- vs. placebo-treated mice. Table S4: Top 50 upregulated and 50 downregulated genes in the oviducts of E2 + P4- vs. placebo-treated mice.

## Abbreviations

The following abbreviations are used in this manuscript:

HGSC	High-grade serous carcinoma
OCs	Oral contraceptives
MPA	Medroxyprogesterone acetate
LD	Progesterone
P4	17β-estradiol
E2	Genetically engineered mouse model
GEMM	Brca1, Trp53, Rb1, and Nf1
BPRN	Tumor suppressor genes
TSGs	Serous tubal intraepithelial carcinomas
STICs	Early high-grade serous carcinoma
eHGSC	Carcinosarcoma
CaSa	Fallopian tube
FT	High-grade serous carcinoma
