# Supplementary material for: Medroxyprogesterone Acetate Inhibits Tumorigenesis in Mouse Models of Oviductal High-Grade Serous Carcinoma
Source: Cancers (Basel). 2025 Oct 28;17(21):3456. doi: 10.3390/cancers17213456 (PMC12610104; doi:10.3390/cancers17213456)
Supplement: Supplementary file 1 [file cancers-17-03456-s001.zip › cancers-3931303-supplementary.pdf]

cancers MDPI

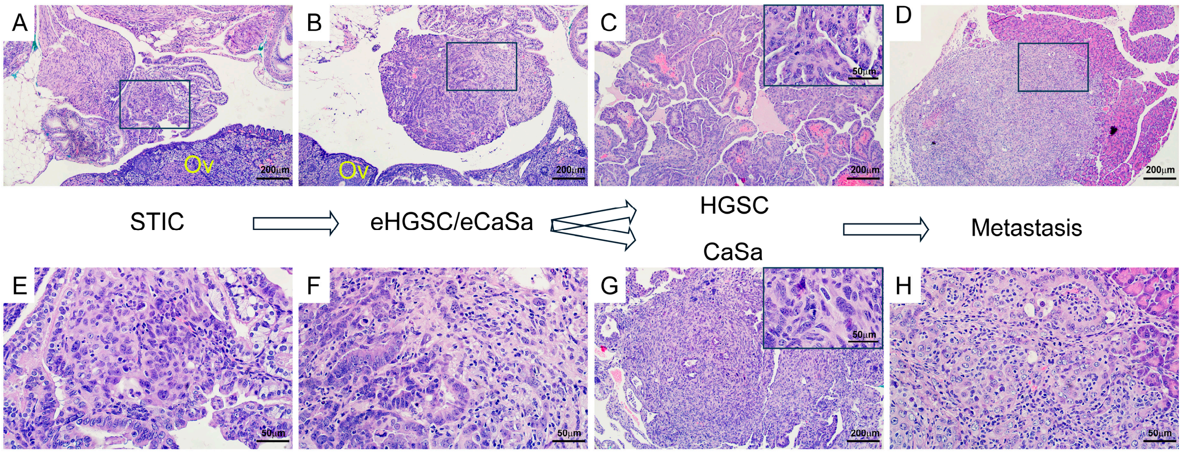


**Supplemental Figure S1.** Representative photomicrographs of H&E-stained sections showing progression of oviductal lesions in *BPRN* mice. Serous tubal intraepithelial carcinoma (STIC, panels A and E), early high-grade serous carcinoma (eHGSC) with focal carcinosarcoma (eCaSa) (panels B, F), HGSC (panel C), CaSa (panel G) and metastases to pancreas (panels D, H) are shown. Scale bars represent 200 μm and 50 μm as indicated.

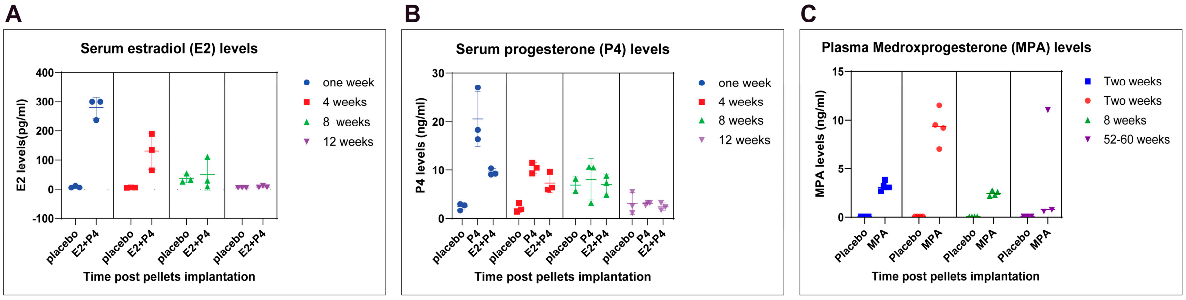


**Supplemental Figure S2.** Serum/plasma hormone levels (E2, P4, MPA) of *Ovgp1-iCreERT2;BPRN* mice at different time points with P4, MPA and E2+P4 treatment.

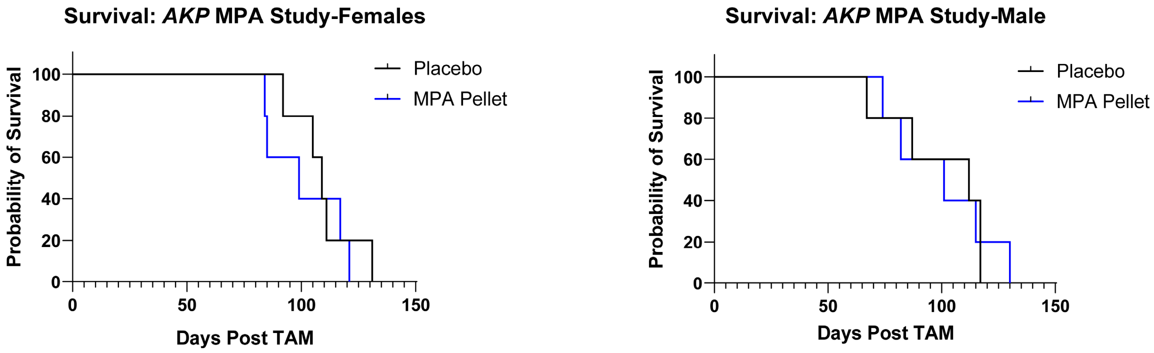


**Supplemetal Figure S3.** Kaplan-Meier survival curves between placebo and MPA implantations in *AKP* mice after TAM injection. Comparison of MPA and placebo mice using the log-rank (Mantel-Cox) test yielded *P* =0.56 for female mice and *P* =0.97 for male mice.

**Table S1.** Hallmark gene sets enriched in differentially expressed genes in the E2+P4, P4, MPA group compared with the Placebo group.

| Gene Set | Description | Size | Expect | Ratio | P Value | FDR |
|---|---|---|---|---|---|---|
| **Upregulated genes (>1.5 FC)** | | | | | | |
| **E2P4_vs_Placebo** | | | | | | |
| HALLMARK_EPITHELIAL_MESENCHY-MAL_TRANSITION | epithelial mesenchymal transition | 200 | 3.0559 | 4.2541 | 0.000007194 | 0.0003597 |
| HALLMARK_MYOGENESIS | muscle differentiation | 200 | 3.0559 | 3.9269 | 0.00003815 | 0.00095376 |
| HALLMARK_HYPOXIA | response to hypoxia; HIF1A targets | 200 | 3.0559 | 2.9451 | 0.0030563 | 0.050938 |
| HALLMARK_COAGULATION | blood coagulation cascade | 138 | 2.1086 | 3.3198 | 0.0047203 | 0.059003 |
| **Downregulated genes (>1.5 FC, FDR ≤ 0.05)** | | | | | | |
| **P4_vs_Placebo** | | | | | | |
| HALLMARK_EPITHELIAL_MESENCHY-MAL_TRANSITION | epithelial mesenchymal transition | 200 | 4.1961 | 4.0514 | 5.55E-07 | 0.000027741 |
| HALLMARK_MTORC1_SIGNALING | mTORC1 signaling | 200 | 4.1961 | 3.3364 | 0.000057692 | 0.0014423 |
| HALLMARK_CHOLESTEROL_HOMEO-STASIS | cholesterol homeostasis | 74 | 1.5526 | 5.1528 | 0.00013392 | 0.002232 |
| HALLMARK_ESTROGEN_RE-SPONSE_EARLY | early estrogen response | 200 | 4.1961 | 3.0981 | 0.00023056 | 0.002882 |
| HALLMARK_HYPOXIA | response to hypoxia; HIF1A targets | 200 | 4.1961 | 2.8598 | 0.00084407 | 0.007034 |
| HALLMARK_ESTROGEN_RE-SPONSE_LATE | late estrogen response | 200 | 4.1961 | 2.8598 | 0.00084407 | 0.007034 |
| HALLMARK_MYOGENESIS | muscle differentiation | 200 | 4.1961 | 2.6215 | 0.0028182 | 0.018426 |
| HALLMARK_UV_RESPONSE_DN | UV response: downregu-lated genes | 144 | 3.0212 | 2.9789 | 0.0029482 | 0.018426 |
| HALLMARK_KRAS_SIGNALING_UP | "KRAS signaling, upregu-lated genes" | 200 | 4.1961 | 2.3832 | 0.0085383 | 0.047435 |
| **Downregulated genes (>1.5 FC)** | | | | | | |
| **MPA_vs_Placebo** | | | | | | |
| HALLMARK_ESTROGEN_RE-SPONSE_LATE | late estrogen response | 200 | 2.919 | 3.7684 | 0.0001195 | 0.0059752 |
| HALLMARK_ESTROGEN_RE-SPONSE_EARLY | early estrogen response | 200 | 2.919 | 3.4258 | 0.00054445 | 0.010838 |
| HALLMARK_CHOLESTEROL_HOMEO-STASIS | cholesterol homeostasis | 74 | 1.08 | 5.5553 | 0.0006503 | 0.010838 |
| HALLMARK_UV_RESPONSE_DN | UV response: downregu-lated genes | 144 | 2.1017 | 3.3306 | 0.004616 | 0.0577 |
| HALLMARK_EPITHELIAL_MESENCHY-MAL_TRANSITION | epithelial mesenchymal transition | 200 | 2.919 | 2.7406 | 0.0079925 | 0.066604 |
| HALLMARK_KRAS_SIGNALING_UP | "KRAS signaling, upregu-lated genes" | 200 | 2.919 | 2.7406 | 0.0079925 | 0.066604 |

**Table S2.** Top 50 upregulated and 50 downregulated genes in the oviducts of P4 vs placebo-treated mice.

| Gene name | log2FC | FC.easy | pval | padj |
|---|---|---|---|---|
| Cacng6 | 5.9112 | 60.1814 | 1.20195E-05 | 0.000552398 |
| Dlk1 | 5.7623 | 54.2766 | 2.05357E-21 | 1.58393E-18 |
| Hoxa11 | 5.2844 | 38.9731 | 0.000363381 | 0.008526957 |
| Actc1 | 4.7879 | 27.6252 | 1.84792E-05 | 0.000773164 |
| Pon1 | 4.5519 | 23.4556 | 1.60218E-05 | 0.000691549 |
| Gm21860 | 3.9888 | 15.8761 | 0.001320575 | 0.022766771 |
| Lrtm2 | 3.6113 | 12.2209 | 0.002291899 | 0.034397869 |
| Tmem132b | 3.3635 | 10.2920 | 4.41861E-20 | 2.90319E-17 |
| Chodl | 3.3445 | 10.1573 | 1.13896E-09 | 1.54238E-07 |
| Adcy8 | 3.2482 | 9.5018 | 7.58681E-07 | 5.09811E-05 |
| Ctla2a | 3.0906 | 8.5182 | 9.32427E-05 | 0.00286677 |
| Il13ra2 | 3.0502 | 8.2833 | 5.48437E-06 | 0.000286999 |
| A730049H05Rik | 3.0381 | 8.2139 | 4.25001E-05 | 0.00148124 |
| Krt23 | 3.0025 | 8.0137 | 9.02783E-16 | 3.40753E-13 |
| Synpr | 2.8901 | 7.4134 | 1.25864E-08 | 1.36148E-06 |
| Cyp2j13 | 2.7721 | 6.8310 | 9.70721E-10 | 1.35595E-07 |
| C7 | 2.7608 | 6.7779 | 2.59619E-05 | 0.001009913 |
| Gm41386 | 2.7035 | 6.5137 | 7.11681E-06 | 0.000354641 |
| Hoxa10 | 2.5616 | 5.9034 | 5.91936E-05 | 0.00197016 |
| Myoc | 2.4575 | 5.4926 | 1.88012E-12 | 4.6324E-10 |
| Prss22 | 2.2280 | 4.6849 | 8.10608E-05 | 0.002558752 |
| Zbtb16 | 2.1212 | 4.3505 | 3.92823E-22 | 3.31842E-19 |
| Lrrtm1 | 2.0172 | 4.0479 | 1.11811E-07 | 9.67571E-06 |
| Ms4a8a | 2.0082 | 4.0229 | 0.002678527 | 0.038320222 |
| Xlr3a | 1.9901 | 3.9727 | 1.40942E-05 | 0.000618889 |
| Brinp2 | 1.9685 | 3.9136 | 0.000172347 | 0.004796902 |
| Coch | 1.9596 | 3.8896 | 3.01932E-13 | 8.50201E-11 |
| Aspg | 1.9348 | 3.8232 | 7.52546E-06 | 0.000370944 |
| Scn4b | 1.9325 | 3.8172 | 3.08176E-09 | 3.77123E-07 |
| Dcstamp | 1.8912 | 3.7095 | 0.000295603 | 0.007273238 |
| Klk1b5 | 1.8466 | 3.5965 | 4.89039E-06 | 0.000258201 |
| Cacng4 | 1.8025 | 3.4883 | 1.14688E-11 | 2.5118E-09 |
| Fzd10os | 1.7734 | 3.4187 | 5.33073E-10 | 7.62638E-08 |
| Cuzd1 | 1.7410 | 3.3426 | 0.001034279 | 0.018898702 |
| Serpina1c | 1.6844 | 3.2140 | 0.001815945 | 0.029163103 |
| Lgi1 | 1.6713 | 3.1850 | 8.99066E-11 | 1.6614E-08 |
| Gm38392 | 1.6638 | 3.1686 | 0.000542343 | 0.011522363 |
| Cacng5 | 1.6362 | 3.1084 | 1.48867E-08 | 1.5909E-06 |
| Gstm7 | 1.6300 | 3.0952 | 7.427E-20 | 4.70554E-17 |
| Zfp648 | 1.6181 | 3.0697 | 9.63028E-06 | 0.000456795 |
| Atp8a2 | 1.6079 | 3.0481 | 3.67255E-06 | 0.000202332 |
| Rhbg | 1.5823 | 2.9944 | 3.42377E-06 | 0.0001904 |
| Gm6721 | 1.5648 | 2.9583 | 0.002203037 | 0.033528301 |
| Krt5 | 1.5578 | 2.9440 | 0.001723643 | 0.028001309 |
| Ptn | 1.5101 | 2.8482 | 4.36227E-09 | 5.19374E-07 |
| Gpx3 | 1.5008 | 2.8300 | 2.56841E-37 | 5.92155E-34 |
| Ctxn3 | 1.4852 | 2.7996 | 0.001153162 | 0.020559901 |
| Folh1 | 1.4557 | 2.7428 | 3.02211E-08 | 2.962E-06 |
| Pla2g5 | 1.4462 | 2.7249 | 3.71057E-18 | 1.93604E-15 |
| Acvr1c | 1.4340 | 2.7019 | 0.000327481 | 0.007861314 |
| Slc38a5 | −1.9585 | −3.8866 | 0.002382763 | 0.035137329 |
| Ldlr | −1.9586 | −3.8870 | 1.78101E-23 | 1.97469E-20 |
| Vwa5b1 | −1.9843 | −3.9567 | 0.000998303 | 0.018409458 |
| Cda | −1.9930 | −3.9807 | 1.91092E-12 | 4.6438E-10 |

| | | | | |
|---|---|---|---|---|
| Alpl | −2.0118 | −4.0328 | 6.70822E-18 | 3.21893E-15 |
| Ifi208 | −2.0310 | −4.0870 | 0.003444311 | 0.046149602 |
| Ldoc1 | −2.0534 | −4.1507 | 0.000532828 | 0.011374684 |
| Lrrc17 | −2.0580 | −4.1642 | 2.75713E-40 | 1.22279E-36 |
| Elfn1 | −2.0746 | −4.2123 | 8.91871E-05 | 0.002761786 |
| Ankle1 | −2.1080 | −4.3111 | 0.002036745 | 0.031694614 |
| Fabp5 | −2.1468 | −4.4285 | 6.9898E-21 | 4.95996E-18 |
| Bean1 | −2.1762 | −4.5195 | 0.001032122 | 0.018895608 |
| Gm6166 | −2.1766 | −4.5209 | 2.62682E-07 | 2.04426E-05 |
| Igf1 | −2.1949 | −4.5786 | 9.61545E-09 | 1.06611E-06 |
| AW551984 | −2.1953 | −4.5797 | 1.06238E-29 | 1.71334E-26 |
| Reg1 | −2.2006 | −4.5967 | 0.002346719 | 0.03489589 |
| Ramp3 | −2.2015 | −4.5995 | 1.68093E-18 | 9.61926E-16 |
| Igkv1-110 | −2.2053 | −4.6118 | 2.25441E-10 | 3.9209E-08 |
| Cd300e | −2.2239 | −4.6716 | 3.80471E-05 | 0.001363547 |
| Gm16010 | −2.2313 | −4.6954 | 2.65638E-15 | 9.42482E-13 |
| 8430419K02Rik | −2.2694 | −4.8214 | 1.57847E-22 | 1.4001E-19 |
| Pappa2 | −2.2870 | −4.8803 | 1.365E-25 | 1.72965E-22 |
| A2m | −2.4274 | −5.3793 | 9.67274E-08 | 8.53703E-06 |
| Igkv1-117 | −2.4382 | −5.4198 | 4.3172E-07 | 3.13882E-05 |
| Gp1bb | −2.4754 | −5.5614 | 1.1263E-38 | 3.99612E-35 |
| Rtl4 | −2.5238 | −5.7511 | 1.02755E-07 | 8.95747E-06 |
| Pde6a | −2.5325 | −5.7858 | 0.000146159 | 0.00419558 |
| Megf11 | −2.6950 | −6.4757 | 1.585E-07 | 1.32632E-05 |
| Lrrc55 | −2.7239 | −6.6064 | 6.63922E-17 | 2.9445E-14 |
| Gm32014 | −2.7460 | −6.7087 | 1.53889E-12 | 3.89999E-10 |
| Ptprq | −2.8238 | −7.0803 | 1.23104E-29 | 1.81989E-26 |
| Ighg2c | −2.8443 | −7.1815 | 0.000331887 | 0.007913548 |
| Dscam | −2.9022 | −7.4759 | 6.01647E-10 | 8.53857E-08 |
| Tmem200a | −2.9310 | −7.6262 | 7.70499E-41 | 4.55622E-37 |
| F5 | −2.9656 | −7.8114 | 2.67037E-37 | 5.92155E-34 |
| Ccl11 | −3.0235 | −8.1311 | 8.47741E-52 | 7.51946E-48 |
| Igkv2-109 | −3.0840 | −8.4799 | 0.003521415 | 0.04693456 |
| Col6a4 | −3.1192 | −8.6891 | 1.01514E-16 | 4.39234E-14 |
| Slc26a4 | −3.2081 | −9.2410 | 6.18626E-08 | 5.77601E-06 |
| Tmprss11g | −3.3290 | −10.0495 | 9.0226E-07 | 5.97242E-05 |
| Ifi206 | −3.5524 | −11.7322 | 4.03293E-06 | 0.000220136 |
| Aqp8 | −3.5991 | −12.1178 | 3.48266E-10 | 5.32607E-08 |
| Nell2 | −3.6641 | −12.6763 | 4.2013E-22 | 3.38777E-19 |
| Khdc1b | −3.8910 | −14.8362 | 0.00203344 | 0.031670967 |
| Cckar | −4.4799 | −22.3141 | 1.18303E-05 | 0.000546536 |
| Lipf | −5.1921 | −36.5583 | 3.13586E-08 | 3.0399E-06 |
| Dmbt1 | −5.3087 | −39.6341 | 2.7546E-07 | 2.13391E-05 |
| Ighv1-81 | −5.3677 | −41.2881 | 1.03006E-07 | 8.95747E-06 |
| Clca1 | −5.5273 | −46.1198 | 0.000168701 | 0.004720435 |
| Krt24 | −6.8272 | −113.5522 | 1.68555E-07 | 1.40383E-05 |

DEGs showed that with fc ≥ 1.5 and padj ≤ 0.05 as cutoff values, a total of 294 up-regulated genens and 403 down-regulated genes were identified in the oviduct of P4 treated mice compared with those of placebo mice .

**Table S3.** Top 50 upregulated and 50 downregulated genes in the oviducts of MPA vs. placebo-treated mice.

| Gene name | log2FC | FC.easy | pval | padj |
|---|---|---|---|---|
| Pon1 | 6.0460 | 66.0737 | 1.97133E-11 | 4.00696E-09 |
| Dlk1 | 5.0512 | 33.1552 | 2.3715E-11 | 4.61256E-09 |
| Cyp2e1 | 4.0663 | 16.7529 | 0.000383006 | 0.009901915 |
| Gm4779 | 3.9091 | 15.0233 | 0.001609732 | 0.030562982 |
| Cfd | 3.8848 | 14.7725 | 0.001559673 | 0.029713721 |
| Plin1 | 3.6987 | 12.9842 | 0.002664375 | 0.044522475 |
| Cidec | 3.5429 | 11.6553 | 0.00124489 | 0.025088284 |
| Chodl | 3.4402 | 10.8541 | 6.2045E-10 | 9.02459E-08 |
| Car3 | 3.3093 | 9.9128 | 0.001891019 | 0.034397977 |
| Fabp4 | 3.2891 | 9.7749 | 0.001913913 | 0.034722359 |
| Tmem132b | 3.1969 | 9.1696 | 7.50194E-12 | 1.69102E-09 |
| B3galt2 | 3.1718 | 9.0117 | 1.03199E-09 | 1.4506E-07 |
| Pck1 | 3.1646 | 8.9670 | 1.18755E-06 | 7.91631E-05 |
| Adrb3 | 3.1113 | 8.6417 | 3.84362E-08 | 3.82691E-06 |
| Slc7a10 | 3.0612 | 8.3464 | 0.000152438 | 0.004644488 |
| Il13ra2 | 3.0368 | 8.2065 | 2.40288E-06 | 0.000145627 |
| Pagr1a | 2.8007 | 6.9677 | 0.000167944 | 0.005007476 |
| C7 | 2.7731 | 6.8357 | 5.56501E-05 | 0.002054878 |
| Synpr | 2.7246 | 6.6098 | 1.4521E-06 | 9.30625E-05 |
| Cuzd1 | 2.6982 | 6.4899 | 1.32299E-06 | 8.57738E-05 |
| Cyp2j13 | 2.6689 | 6.3595 | 1.07727E-09 | 1.48922E-07 |
| Krt23 | 2.6439 | 6.2501 | 1.59632E-11 | 3.37995E-09 |
| Gm41386 | 2.5859 | 6.0038 | 2.31824E-05 | 0.001017773 |
| Mrap | 2.5683 | 5.9312 | 5.06794E-05 | 0.001926625 |
| Cd36 | 2.5456 | 5.8385 | 4.57767E-05 | 0.001780713 |
| Cdo1 | 2.4446 | 5.4436 | 0.000562292 | 0.0136311 |
| Abcd2 | 2.3237 | 5.0061 | 4.84417E-12 | 1.12539E-09 |
| Lpl | 2.3213 | 4.9978 | 9.54484E-05 | 0.003199528 |
| Atp8a2 | 2.3203 | 4.9942 | 6.12093E-21 | 5.11924E-18 |
| Acvr1c | 2.2664 | 4.8111 | 7.58215E-12 | 1.69102E-09 |
| A730046J19Rik | 2.2098 | 4.6262 | 6.63338E-05 | 0.002365813 |
| Aspg | 2.1599 | 4.4689 | 2.50707E-07 | 2.02588E-05 |
| Orm1 | 2.0909 | 4.2602 | 2.40359E-05 | 0.001036208 |
| Fgf1 | 2.0901 | 4.2577 | 9.9668E-86 | 1.66715E-81 |
| Lgals12 | 2.0113 | 4.0314 | 0.000106726 | 0.003479936 |
| Folh1 | 1.9919 | 3.9776 | 2.22043E-14 | 7.57983E-12 |
| Myoc | 1.9660 | 3.9068 | 2.55988E-05 | 0.001084466 |
| Zbtb16 | 1.9244 | 3.7958 | 3.51938E-16 | 1.50945E-13 |
| Lrrtm1 | 1.8851 | 3.6939 | 1.38203E-05 | 0.00068394 |
| Dcstamp | 1.8398 | 3.5797 | 0.000367772 | 0.009672521 |
| Coch | 1.8075 | 3.5003 | 2.66309E-09 | 3.36298E-07 |
| Rarres1 | 1.7869 | 3.4506 | 0.000378695 | 0.009851361 |
| Apol6 | 1.7790 | 3.4320 | 0.00026258 | 0.007188499 |
| Trank1 | 1.7597 | 3.3862 | 3.07461E-21 | 2.70679E-18 |
| Cacng4 | 1.7499 | 3.3634 | 5.9474E-10 | 8.80374E-08 |
| Scn4b | 1.7465 | 3.3555 | 4.46188E-07 | 3.39245E-05 |
| C030017B01Rik | 1.7272 | 3.3109 | 0.000132106 | 0.004122656 |
| Aldh1a7 | 1.6770 | 3.1976 | 0.000319638 | 0.008527257 |
| Zfp648 | 1.6639 | 3.1687 | 0.000230018 | 0.006437093 |
| Gstm7 | 1.5612 | 2.9510 | 2.37065E-24 | 3.0503E-21 |
| Pimreg | −1.6728 | −3.1883 | 0.000378283 | 0.009851361 |
| A2m | −1.6731 | −3.1891 | 6.10169E-07 | 4.43752E-05 |
| Gm32014 | −1.7198 | −3.2938 | 0.000350649 | 0.009280541 |
| Igkv1-117 | −1.7222 | −3.2993 | 1.8818E-11 | 3.88604E-09 |

| | | | | |
|---|---|---|---|---|
| Igkv1-110 | −1.7227 | −3.3006 | 1.65495E-05 | 0.000793191 |
| Dsg3 | −1.7361 | −3.3313 | 0.004304046 | 0.062603281 |
| Ldoc1 | −1.8455 | −3.5937 | 0.003089426 | 0.049641522 |
| Mgam | −1.8517 | −3.6093 | 0.002259436 | 0.039574434 |
| Alpl | −1.8529 | −3.6122 | 5.63841E-17 | 2.9473E-14 |
| 9130019P16Rik | −1.8552 | −3.6179 | 2.32825E-11 | 4.58172E-09 |
| Cd300e | −1.8637 | −3.6394 | 0.000196519 | 0.005628729 |
| Igf1 | −1.8912 | −3.7094 | 5.33916E-18 | 3.18957E-15 |
| Ramp3 | −1.8921 | −3.7118 | 4.36266E-11 | 7.6815E-09 |
| Gm9902 | −1.9169 | −3.7762 | 0.001003979 | 0.021230796 |
| Utf1 | −1.9401 | −3.8374 | 0.000492398 | 0.012220083 |
| Krt12 | −1.9670 | −3.9096 | 1.84096E-08 | 1.92461E-06 |
| Gpr165 | −1.9986 | −3.9960 | 0.000333438 | 0.008853035 |
| Prss29 | −2.0133 | −4.0369 | 0.000742337 | 0.016963205 |
| Gm16010 | −2.0135 | −4.0376 | 2.26671E-16 | 1.02473E-13 |
| Pde6a | −2.0741 | −4.2107 | 0.000123501 | 0.003927368 |
| Krt83 | −2.1119 | −4.3226 | 6.0644E-10 | 8.89817E-08 |
| Igkv1-135 | −2.1553 | −4.4545 | 0.001349084 | 0.026579203 |
| Tmem200a | −2.1872 | −4.5542 | 1.35224E-13 | 4.26773E-11 |
| Igkv10-96 | −2.1905 | −4.5645 | 0.000124885 | 0.003956018 |
| Ccl11 | −2.1977 | −4.5873 | 8.39596E-22 | 8.26113E-19 |
| Col6a4 | −2.2638 | −4.8026 | 4.3861E-05 | 0.001722212 |
| Gad2 | −2.2722 | −4.8306 | 6.20096E-09 | 7.10434E-07 |
| Ptprq | −2.2941 | −4.9046 | 1.91088E-22 | 1.99771E-19 |
| Igkv12-44 | −2.3186 | −4.9886 | 0.002193479 | 0.038784704 |
| Ptpn5 | −2.3425 | −5.0719 | 9.60474E-21 | 7.65041E-18 |
| Igkv14-111 | −2.3511 | −5.1020 | 0.001465736 | 0.028409466 |
| AW551984 | −2.3894 | −5.2396 | 1.77565E-25 | 2.70012E-22 |
| Gp1bb | −2.3970 | −5.2672 | 5.00764E-15 | 1.78219E-12 |
| Lrrc55 | −2.3995 | −5.2760 | 1.56336E-16 | 7.37552E-14 |
| Igha | −2.4488 | −5.4598 | 0.000155498 | 0.004694977 |
| Aipl1 | −2.5887 | −6.0154 | 1.64244E-43 | 1.37365E-39 |
| Megf11 | −2.6242 | −6.1655 | 1.99395E-07 | 1.66887E-05 |
| F5 | −2.6988 | −6.4926 | 2.78135E-30 | 5.16929E-27 |
| Elfn1 | −2.7831 | −6.8835 | 7.89999E-11 | 1.3623E-08 |
| Dscam | −2.9167 | −7.5512 | 1.88092E-11 | 3.88604E-09 |
| Krt24 | −2.9531 | −7.7441 | 2.88913E-06 | 0.000168974 |
| Nell2 | −3.2036 | −9.2124 | 2.02044E-18 | 1.31776E-15 |
| Igkv2-137 | −3.4700 | −11.0809 | 6.94177E-06 | 0.000378225 |
| Slc26a4 | −3.5337 | −11.5809 | 2.67398E-09 | 3.36298E-07 |
| Tmprss11g | −3.5984 | −12.1121 | 4.19557E-11 | 7.54616E-09 |
| Clca1 | −3.6050 | −12.1678 | 0.001473626 | 0.028463441 |
| Aqp8 | −3.9983 | −15.9810 | 2.18867E-37 | 7.32199E-34 |
| Dmbt1 | −5.8899 | −59.2976 | 9.65046E-08 | 8.71636E-06 |
| Serpina1e | −5.9252 | −60.7678 | 0.003061169 | 0.049282166 |
| Ighv1-66 | −7.1146 | −138.5781 | 7.03508E-07 | 5.02888E-05 |
| Lipf | −7.2572 | −152.9841 | 2.25691E-08 | 2.30191E-06 |

DEGs showed that with fc ≥ 1.5 and padj ≤ 0.05 as cutoff values, a total of 284 up-regulated genens and 302 down-regulated genes were identified in the oviduct of MPA treated mice compared with those of placebo mice.

**Table S4.** Top 50 upregulated and 50 downregulated genes in the oviducts of E2+P4 vs. placebo-treated mice.

| Gene name | log2FC | FC.easy | pval | padj |
|---|---|---|---|---|
| Cstdc2 | 6.1571 | 71.3617 | 0.000252927 | 0.010846671 |
| Hoxa11 | 5.2656 | 38.4685 | 0.000266496 | 0.011300733 |
| A730049H05Rik | 4.8356 | 28.5528 | 1.13674E-14 | 7.18229E-12 |
| Actc1 | 4.6917 | 25.8426 | 2.53025E-05 | 0.001671109 |
| Adcy8 | 4.6427 | 24.9806 | 3.15598E-13 | 1.49554E-10 |
| Lrtm2 | 4.2570 | 19.1202 | 1.54117E-06 | 0.000153752 |
| Dlk1 | 4.1084 | 17.2483 | 5.51078E-08 | 8.56203E-06 |
| Tmem132b | 4.1034 | 17.1884 | 1.16948E-26 | 2.21675E-23 |
| Ctla2a | 3.9104 | 15.0366 | 6.20306E-54 | 1.17579E-49 |
| C7 | 3.8437 | 14.3567 | 3.69008E-14 | 1.99778E-11 |
| Il13ra2 | 3.3770 | 10.3890 | 1.55415E-07 | 2.08928E-05 |
| Chodl | 3.2481 | 9.5011 | 9.72765E-06 | 0.000768282 |
| Prss22 | 3.1933 | 9.1473 | 1.03178E-05 | 0.000803857 |
| Dpep1 | 3.1537 | 8.8992 | 2.95121E-06 | 0.000274217 |
| Brinp2 | 3.1038 | 8.5970 | 2.99119E-14 | 1.66759E-11 |
| Krt4 | 3.0904 | 8.5173 | 2.91793E-05 | 0.001887691 |
| Hapln4 | 3.0536 | 8.3030 | 8.48226E-06 | 0.000678402 |
| Mcpt4 | 3.0306 | 8.1716 | 9.74751E-05 | 0.005118117 |
| Krt23 | 2.9524 | 7.7405 | 4.89866E-12 | 1.85708E-09 |
| Bco1 | 2.9301 | 7.6214 | 3.89529E-09 | 7.93927E-07 |
| Rgs1 | 2.7929 | 6.9301 | 0.001729071 | 0.048268831 |
| Hoxa10 | 2.7842 | 6.8883 | 2.14563E-05 | 0.001484323 |
| Cuzd1 | 2.7117 | 6.5508 | 1.62757E-06 | 0.00016068 |
| Trnp1 | 2.5910 | 6.0253 | 1.3258E-05 | 0.000977842 |
| Cyp2j13 | 2.5638 | 5.9124 | 6.13852E-07 | 6.92593E-05 |
| Tpsb2 | 2.5499 | 5.8559 | 0.000621568 | 0.022484377 |
| Astn1 | 2.5082 | 5.6890 | 1.55277E-14 | 9.19776E-12 |
| Hsd11b2 | 2.4918 | 5.6248 | 6.46879E-11 | 2.12358E-08 |
| Plcxd3 | 2.4799 | 5.5785 | 0.000481456 | 0.01832528 |
| Myom2 | 2.4710 | 5.5442 | 0.00024059 | 0.010422032 |
| Myoc | 2.4008 | 5.2811 | 1.69836E-05 | 0.001219411 |
| Msmp | 2.3632 | 5.1450 | 0.001038171 | 0.03341007 |
| Cma1 | 2.3559 | 5.1191 | 0.000773362 | 0.026976256 |
| Cacng4 | 2.3182 | 4.9872 | 6.49649E-10 | 1.59923E-07 |
| Zbtb16 | 2.2705 | 4.8250 | 7.19555E-17 | 5.93007E-14 |
| Krt79 | 2.2497 | 4.7559 | 4.81823E-08 | 7.67475E-06 |
| Klk1b1 | 2.2396 | 4.7225 | 0.001484145 | 0.043279953 |
| Synpr | 2.1942 | 4.5763 | 8.63338E-05 | 0.004688989 |
| P2rx5 | 2.1890 | 4.5599 | 9.28031E-05 | 0.004941244 |
| Aspg | 2.0926 | 4.2651 | 8.42982E-11 | 2.61946E-08 |
| Pla1a | 2.0694 | 4.1970 | 2.64429E-10 | 7.37096E-08 |
| Ajap1 | 2.0684 | 4.1942 | 0.001666586 | 0.047149459 |
| Xlr3a | 2.0550 | 4.1554 | 1.86493E-07 | 2.47402E-05 |
| Atp8a2 | 2.0441 | 4.1242 | 4.67169E-12 | 1.80718E-09 |
| Scn4b | 2.0241 | 4.0674 | 9.25637E-07 | 9.85699E-05 |
| Cpxm2 | 2.0229 | 4.0641 | 5.13756E-22 | 5.72838E-19 |
| Plac9b | 1.9759 | 3.9339 | 1.36819E-09 | 3.08739E-07 |
| Gpx3 | 1.9303 | 3.8114 | 1.00901E-48 | 9.56294E-45 |
| Gm9780 | 1.8944 | 3.7177 | 1.23684E-06 | 0.000126045 |
| Capn6 | 1.8746 | 3.6670 | 7.9463E-15 | 5.19387E-12 |
| Pappa2 | −1.9362 | −3.8269 | 3.16741E-17 | 2.85897E-14 |
| Megf11 | −2.0041 | −4.0115 | 0.000141834 | 0.00696492 |
| Nat8f3 | −2.0129 | −4.0358 | 0.000257192 | 0.01097989 |
| Col6a4 | −2.0842 | −4.2404 | 1.33551E-14 | 8.16599E-12 |

| | | | | |
|---|---|---|---|---|
| Grem2 | −2.0854 | −4.2439 | 9.98841E-47 | 6.31101E-43 |
| Lrrc17 | −2.1714 | −4.5047 | 4.21906E-33 | 1.59944E-29 |
| Nell2 | −2.2446 | −4.7390 | 1.21384E-10 | 3.53975E-08 |
| Igkc | −2.2644 | −4.8046 | 8.93555E-05 | 0.004811743 |
| Igkv1-135 | −2.2838 | −4.8694 | 0.000135221 | 0.006709743 |
| Esr2 | −2.2844 | −4.8716 | 0.001626761 | 0.046438636 |
| Apod | −2.3398 | −5.0623 | 6.40087E-07 | 7.13697E-05 |
| F5 | −2.4214 | −5.3567 | 3.79524E-22 | 4.49618E-19 |
| Pck1 | −2.4214 | −5.3569 | 0.000134665 | 0.006699695 |
| Col6a6 | −2.4305 | −5.3908 | 7.9695E-11 | 2.56037E-08 |
| Necab2 | −2.4675 | −5.5309 | 4.80527E-13 | 2.16866E-10 |
| Igkv1-117 | −2.5736 | −5.9529 | 3.27384E-06 | 0.000299786 |
| Igha | −2.6531 | −6.2901 | 0.001085809 | 0.034359786 |
| Iglv1 | −2.6704 | −6.3661 | 0.000721917 | 0.025482202 |
| Ccl11 | −2.6809 | −6.4124 | 2.74672E-33 | 1.3016E-29 |
| Slc38a5 | −2.6894 | −6.4503 | 5.60101E-07 | 6.53853E-05 |
| Ighg2c | −2.6895 | −6.4509 | 0.001199358 | 0.036845743 |
| 9130019P16Rik | −2.7040 | −6.5161 | 2.30183E-25 | 3.96647E-22 |
| Ptprq | −2.7169 | −6.5745 | 1.35198E-22 | 1.70845E-19 |
| Jchain | −2.7756 | −6.8475 | 9.9119E-06 | 0.000779585 |
| Igkv5-43 | −2.8435 | −7.1775 | 0.001196443 | 0.036815857 |
| A2m | −2.8644 | −7.2825 | 2.51289E-08 | 4.41036E-06 |
| A230077H06Rik | −2.9534 | −7.7460 | 0.000563235 | 0.020770664 |
| Igkv15-103 | −3.0472 | −8.2661 | 0.000485908 | 0.018384007 |
| Igkv2-137 | −3.0939 | −8.5382 | 0.000162112 | 0.007688773 |
| Igkv14-111 | −3.1951 | −9.1586 | 2.46188E-05 | 0.001637363 |
| Lrrc55 | −3.3161 | −9.9599 | 1.93832E-23 | 2.82622E-20 |
| Slc26a4 | −3.3167 | −9.9635 | 1.32719E-06 | 0.000133813 |
| Slc38a3 | −3.4425 | −10.8714 | 1.48743E-05 | 0.001084396 |
| Igkv12-44 | −3.5480 | −11.6967 | 8.1764E-07 | 8.9071E-05 |
| Cckar | −3.5780 | −11.9422 | 1.55696E-05 | 0.001122134 |
| Igkv6-23 | −3.6977 | −12.9753 | 0.000598859 | 0.021829552 |
| Igkv10-96 | −3.7882 | −13.8156 | 4.91862E-08 | 7.76937E-06 |
| Ighv1-64 | −3.8538 | −14.4583 | 0.001333934 | 0.039693433 |
| Ighv1-66 | −3.9675 | −15.6439 | 3.85882E-05 | 0.002398162 |
| Gm13716 | −4.1654 | −17.9435 | 0.001401314 | 0.0412452 |
| Krt24 | −4.2949 | −19.6285 | 5.7569E-06 | 0.000489336 |
| Igkv19-93 | −4.3199 | −19.9721 | 0.001651584 | 0.046864934 |
| Nxph2 | −4.3795 | −20.8138 | 0.001203912 | 0.036925799 |
| Igkv6-25 | −4.5639 | −23.6523 | 6.77886E-05 | 0.003847105 |
| Dmbt1 | −4.8762 | −29.3691 | 1.01457E-06 | 0.000106112 |
| Igkv17-121 | −5.1935 | −36.5924 | 0.000817327 | 0.027964675 |
| Krt26 | −6.2068 | −73.8640 | 9.07492E-05 | 0.00487295 |
| Igkv9-124 | −6.5073 | −90.9685 | 0.00018018 | 0.008330011 |
| Igkv12-41 | −6.5604 | −94.3797 | 0.000175368 | 0.008147286 |
| Igkv2-109 | −6.9134 | −120.5428 | 3.02905E-06 | 0.000280076 |

DEGs showed that with fc ≥ 1.5 and padj ≤ 0.05 as cutoff values, a total of 272 up-regulated genens and 242 down-regulated genes were identified in the oviduct of E2+P4 treated mice compared with those of placebo mice.
